# Supplementary figures and images for: Insurance instability and use of emergency and office-based care after gaining coverage: An observational cohort study
Source: PLoS One. 2020 Sep 4;15(9):e0238100. doi: 10.1371/journal.pone.0238100 (PMC7473517; doi:10.1371/journal.pone.0238100)

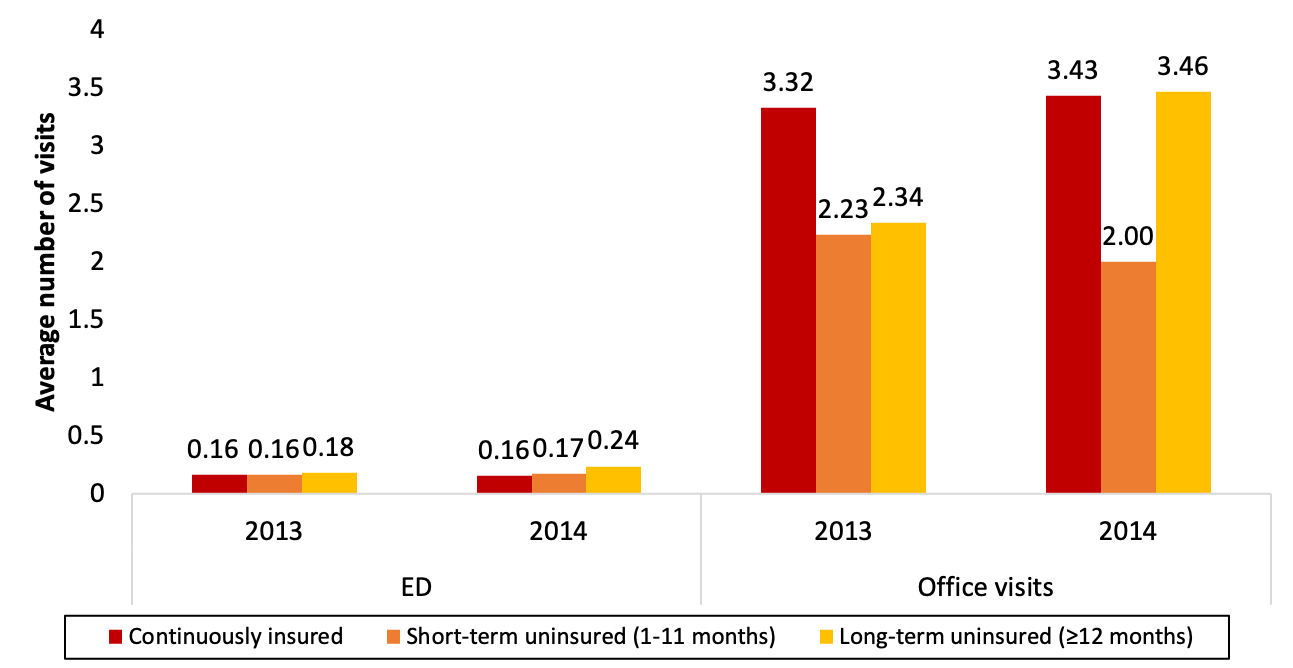

Supplement: S1 Fig — N = 6,371. (TIF) [file pone.0238100.s001.tif]
