# Supplementary material for: Insurance instability and use of emergency and office-based care after gaining coverage: An observational cohort study
Source: PLoS One. 2020 Sep 4;15(9):e0238100. doi: 10.1371/journal.pone.0238100 (PMC7473517; doi:10.1371/journal.pone.0238100)
Supplement: S2 Table — (DOCX) [file pone.0238100.s003.docx]

**S2 Table. Weighted linear regression estimates of year-over-year change in office visits, United States, 2013–2014**

| **Change in office visits** | *Coefficient (standard error)* | | | |
| --- | --- | --- | --- | --- |
|  | (1) | (2) | (3) | (4) |
| *Insurance instability  prior to 2014* |  |  |  |  |
| Continuously insured | – | – | – | – |
| Short-term uninsured | –0.34 (0.31) | –0.15 (0.36) | –0.24 (0.36) | –0.21 (0.36) |
| Long-term uninsured | 1.02* (0.50) | 1.24* (0.60) | 1.22* (0.60) | 1.18 (0.61) |
| *Controls* |  |  |  |  |
| Individual-level |  | X | X | X |
| County-level |  |  | X | X |
| State fixed effects |  |  |  | X |
| Number of observations | 6,435 | 6,371 | 6,371 | 6,371 |

* p<0.05, ** p<0.01

Individual-level controls include age, sex, race/ethnicity, education, employment status, marital status, household size, household income, presence of a perceived decline in health from 2013 to 2014, and presence of an ambulatory care sensitive condition. County-level controls include number of hospitals with an ED (quartiles), number of primary care physicians (quartiles), number of physician extenders (quartiles), number of federally qualified health centers (quartiles), percentage of population non-white, percentage of population unemployed, percentage of population in poverty, percentage of population uninsured, being in a health professional shortage area, and non-metro area.
