# Supplementary material for: Insurance instability and use of emergency and office-based care after gaining coverage: An observational cohort study
Source: PLoS One. 2020 Sep 4;15(9):e0238100. doi: 10.1371/journal.pone.0238100 (PMC7473517; doi:10.1371/journal.pone.0238100)
Supplement: S5 Table — (DOCX) [file pone.0238100.s006.docx]

**S5 Table. Sensitivity analysis of year-over-year change in office visits,
United States, 2013–2014**

| **Change in office visits** | *Coefficient (standard error)* | | | |
| --- | --- | --- | --- | --- |
|  | Full analytic sample | Drop ages  18 to 26 | Drop those >400% FPL | Include with >0 total visits in both years |
| *Insurance instability  prior to 2014* |  |  |  |  |
| Continuously insured | – | – | – | – |
| Short-term uninsured | –0.21 (0.36) | –0.47 (0.39) | –0.24 (0.39) | –0.67 (0.59) |
| Long-term uninsured | 1.18 (0.61) | 0.92* (0.37) | 0.65* (0.32) | 2.62 (1.45) |
| Number of observations | 6,371 | 5,205 | 4,147 | 3,549 |

* p<0.05, ** p<0.01

All models are weighted and include individual- and county-level controls and state fixed effects.
