# Supplementary material for: Insurance instability and use of emergency and office-based care after gaining coverage: An observational cohort study
Source: PLoS One. 2020 Sep 4;15(9):e0238100. doi: 10.1371/journal.pone.0238100 (PMC7473517; doi:10.1371/journal.pone.0238100)
Supplement: S6 Table — (DOCX) [file pone.0238100.s007.docx]

**S6 Table. Sensitivity analysis of year-over-year substitution, United States, 2013–2014**

| **Substitution** | *Coefficient (standard error)* | | | |
| --- | --- | --- | --- | --- |
|  | Full analytic sample | Drop ages  18 to 26^†^ | Drop those >400% FPL | Include with >0 total visits in both years |
| *Outcome categories* |  |  |  |  |
| > ED visits, > office visits | | | | |
| Continuously insured | – | – | – | – |
| Short-term uninsured | –0.06 (0.41) | –0.10 (–) | –0.04 (0.52) | –0.28 (0.63) |
| Long-term uninsured | 0.25 (0.22) | 0.23 (–) | 0.54* (0.24) | 0.39 (0.34) |
| ≤ ED visits, > office visits | | | | |
| Continuously insured | – | – | – | – |
| Short-term uninsured | –0.04 (0.19) | –0.14 (–) | 0.15 (0.20) | 0.02 (0.23) |
| Long-term uninsured | 0.05 (0.14) | 0.06 (–) | 0.11 (0.14) | 0.32 (0.20) |
| > ED visits, ≤ office visits | | | | |
| Continuously insured | – | – | – | – |
| Short-term uninsured | 0.73* (0.37) | 1.09 (–) | 0.76 (0.41) | 0.55 (0.40) |
| Long-term uninsured | 0.16 (0.31) | –0.07 (–) | 0.34 (0.29) | 0.31 (0.37) |
| ≤ ED visits, ≤office visits *(base outcome)* | | | | |
| Number of observations | 6,371 | 5,205 | 4,147 | 3,549 |

^†^ Weighted variance matrix was nonsymmetric, standard errors and p-values not obtained.

* p<0.05, ** p<0.01

All models are weighted and include individual- and county-level controls and state fixed effects.
