# Supplementary material for: Insurance instability and use of emergency and office-based care after gaining coverage: An observational cohort study
Source: PLoS One. 2020 Sep 4;15(9):e0238100. doi: 10.1371/journal.pone.0238100 (PMC7473517; doi:10.1371/journal.pone.0238100)
Supplement: Attachment — Submitted filename: Response to Reviewers_R1 v2.docx [file pone.0238100.s008.docx]

**Insurance instability and use of emergency and office-based care
after gaining coverage: an observational cohort study**

Response to Reviewers

**Editors**

1) Please ensure that your manuscript meets PLOS ONE's style requirements, including those for file naming. The PLOS ONE style templates can be found at <https://journals.plos.org/plosone/s/file?id=wjVg/PLOSOne_formatting_sample_main_body.pdf>

and <https://journals.plos.org/plosone/s/file?id=ba62/PLOSOne_formatting_sample_title_authors_affiliations.pdf>.

**AUTHOR RESPONSE**: We have made several minor updates to bring the manuscript in line with the journal style requirements.

2) We note that you have indicated that data from this study are available upon request. PLOS only allows data to be available upon request if there are legal or ethical restrictions on sharing data publicly. For information on unacceptable data access restrictions, please see [http://journals.plos.org/plosone/s/data-availability - loc-unacceptable-data-access-restrictions](http://journals.plos.org/plosone/s/data-availability#loc-unacceptable-data-access-restrictions)

In your revised cover letter, please address the following prompts:

a) If there are ethical or legal restrictions on sharing a de-identified data set, please explain them in detail (e.g., data contain potentially identifying or sensitive patient information) and who has imposed them (e.g., an ethics committee). Please also provide contact information for a data access committee, ethics committee, or other institutional body to which data requests may be sent.

b) If there are no restrictions, please upload the minimal anonymized data set necessary to replicate your study findings as either Supporting Information files or to a stable, public repository and provide us with the relevant URLs, DOIs, or accession numbers. Please see <http://www.bmj.com/content/340/bmj.c181.long> for guidelines on how to de-identify and prepare clinical data for publication. For a list of acceptable repositories, please see <http://journals.plos.org/plosone/s/data-availability#loc-recommended-repositories>.

We will update your Data Availability statement on your behalf to reflect the information you provide.

**AUTHOR RESPONSE**: The following clarification on data availability is reflected in our revised cover letter and also included here for ease of reference in updating our Data Availability statement.

*“This study is based on the publicly available Medical Expenditure Panel (MEPS) Survey Panel 18 Longitudinal Data File (*[*https://meps.ahrq.gov/mepsweb/data_stats/download_data_files_detail.jsp?cboPufNumber=HC-172*](https://meps.ahrq.gov/mepsweb/data_stats/download_data_files_detail.jsp?cboPufNumber=HC-172)*) and 2012 National Health Interview Survey (NHIS) Sample Adult File (*[*https://www.cdc.gov/nchs/nhis/nhis_2012_data_release.htm*](https://www.cdc.gov/nchs/nhis/nhis_2012_data_release.htm)*). The linkage of respondent identifiers between these two data sets as well as the state and county identifiers used to merge on county-level characteristics are only available upon approval of a restricted data use proposal to the Agency for Healthcare Research and Quality (AHRQ) for analysis in a Federal Statistical Research Data Center (RDC). AHRQ provides a description about the confidential and non-public use variables available in the RDC (*[*https://meps.ahrq.gov/mepsweb/data_stats/onsite_datacenter.jsp*](https://meps.ahrq.gov/mepsweb/data_stats/onsite_datacenter.jsp)*) and documentation for the NHIS-MEPS linkage (*[*https://meps.ahrq.gov/mepsweb/data_stats/download_data_files_detail.jsp?cboPufNumber=NHIS%20Link*](https://meps.ahrq.gov/mepsweb/data_stats/download_data_files_detail.jsp?cboPufNumber=NHIS%20Link)*) on their web site. Proposals for access to restricted variables (*[*https://meps.ahrq.gov/data_stats/data_center_application.pdf*](https://meps.ahrq.gov/data_stats/data_center_application.pdf)*) should be submitted via email to* [*cfactdc@ahrq.hhs.gov*](mailto:cfactdc@ahrq.hhs.gov)*.”*

3) Please confirm that references conform to PLOS ONE requirements. For example, journal abbreviations should not include periods, i.e., use "N Engl J Med" instead of "N. Engl. J. Med". For further information, refer to: <https://journals.plos.org/plosone/s/submission-guidelines#loc-references>

**AUTHOR RESPONSE**: We have removed the extra periods in the journal abbreviations and made several updates to webpage references to meet the reference formatting requirements.

**Reviewer 1**

Thank you for the opportunity to review this paper. Using data from the Medical Expenditure Panel Survey (MEPS), the authors investigate the association between prior ‘insurance stability’ and change in use of emergency and office visits after the introduction of Medicaid expansion and Marketplace. The policy significance of this research question is obvious. The authors have used an appropriate data set and appropriate methods for their analysis.

1) However, the major issue with this paper is whether it makes sufficiently new contribution to the literature. The authors claim that “However, the relationship between how long someone went uninsured before gaining coverage, what we refer to as insurance instability, and subsequent changes in health care use after gaining coverage has not yet been studied” (lines 64-67). Unfortunately it’s not quite true. Kirby and Vistnes (2016) answered a very similar research question using the same data set and similar methods. The following is the abstract of that paper, from Health Affairs (<https://www.healthaffairs.org/doi/10.1377/hlthaff.2016.0716>

“Newly available longitudinal survey data show that people who lacked health insurance in 2013 and gained coverage through Medicaid or the Marketplaces in 2014 were far more likely to obtain a usual source of care and receive preventive care services than their counterparts who remained uninsured in 2014.”

Since this paper was not in the citations, I assume that the authors are not aware of this research. The only thing that seems to be different in this paper from the Health Affairs paper is that the authors are using different measures of health care use, though preventive service visits are likely a subset of office visits.

The authors therefore need to do a more careful review of the literature - incorporate the above-mentioned paper and make sure that they haven’t missed any other relevant study. They also need to convince the readers that their research in not just a minor extension of existing research.

**AUTHOR RESPONSE**: Thank you for the suggestion, we have added this paper to the literature cited in our manuscript. That paper looks at a closely related but different question to ours. Kirby and Vistnes (2016) focused on those uninsured for all of 2013, comparing outcomes among those gaining coverage in 2014 to those that remained uninsured, which provided important insight into how gaining coverage affects access to care for the uninsured. Meanwhile, we examine how past insurance instability affects health care use after gaining coverage, comparing how pre-existing differences in use of ED and office-based care across these groups (continuously insured, short-term uninsured, long-term uninsured) change once the latter two groups gain coverage. They use a different comparison group and drop those with only partial year coverage in 2013, which we do not. In fact, looking at the differences in outcomes for partial (short-term) and full-year (long-term) uninsured is central to our study. Also, their study was descriptive in nature only and did not include any adjusted regression analyses or visit-based utilization outcomes. We say that not to diminish their work in any way but to highlight that we believe our study makes a separate and worthwhile contribution to the literature.

We have also added citations and appropriate discussion of a six additional papers on insurance instability, including two published in the last few months, and its effects to bolster our argument as to why this is an important health policy issue in both the Introduction (on page 4) and the Discussion (on page 17). Of the newly cited studies that relate insurance instability to health care use, all were published using pre-ACA study periods. We have included the new text below for ease of reference.

**Introduction** (bottom of page 4)

*“Longer periods of being uninsured has been associated with lower access to care and those with unstable coverage are more likely to use the emergency department as a primary source of care, refill prescriptions less frequently, and have uncontrolled hypertension [16-20]. Disparities in insurance instability by race and ethnicity help compound difficulties in access to care and help maintain health inequities, though there is evidence of reduced Hispanic insurance instability post-ACA [20,21].”*

18.	Banerjee R, Ziegenfuss JY, Shah ND. Impact of discontinuity in health insurance on resource utilization. BMC Health Serv Res. 2010;10:195. doi: 10.1186/1472-6963-10-195.

19.	Fields BE, Bell JF, Moyce S, Bigbee JL. The impact of insurance instability on health service utilization: does non-metropolitan residence make a difference? J Rural Health. 2015;31(1):27-34. doi: 10.1111/jrh.12077.

20.	Kressin NR, Terrin N, Hanchate AD, Price LL, Moreno-Koehler A, LeClair A, Suzukida J, Kher S, Freund KM. Is insurance instability associated with hypertension outcomes and does this vary by race/ethnicity? BMC Health Serv Res. 2020;20(1):216. doi: 10.1186/s12913-020-05095-8.

21.	Gai Y, Jones K. Insurance patterns and instability from 2006 to 2016. BMC Health Serv Res. 2020;20(1):334. doi: 10.1186/s12913-020-05226-1.

**Discussion** (second paragraph on page 17)

*“Prior expansions, such as Massachusetts health reform, have increased the insured rate without increasing the frequency of plan switches [35], another dimension of instability that can be related to access barriers, and reducing coverage gaps for some populations [36].”*

35.	Freund KM, Isabelle AP, Hanchate A, Kalish RL, Kapoor A, Bak S, Mishuris RG, Shroff S, Battaglia TA. The Impact of Health Insurance Reform on Insurance Instability. J Health Care Poor Undeserved. 2014;25(1):95-108. doi: 10.1353/hpu.2014.0061

36.	Freund KM, LeClair A, Terrin N, Hanchate AD, Price LL, Moreno-Koehler A, Suzukida J, Kher S, Byhoff E, Kressin NR. Racial Differences in Insurance Stability after Health Insurance Reform. Med Care. 2019;57(4):256-261. doi: 10.1097/MLR.0000000000001078

2) The authors should exclude the 18-year-olds from the sample. The 18-year-olds have public coverage options from Medicaid or Children’s Health Insurance Program (CHIP) as children that are more generous than those for adults (even in the expansion states).

**AUTHOR RESPONSE**: I appreciate this point from the reviewer and generally agree that this minor change would be useful. However, the COVID-19 pandemic has made any re-analysis of our data impossible for the time being. The Census Bureau has closed all of its Federal Statistical Research Data Centers indefinitely. The administrator at the Boston RDC, where I have access to these restricted data, has said that it will be at least September before they reopen and potentially longer based on how the pandemic evolves during the summer. In light of this, I have used the public use MEPS data to reconstruct our analytic sample to address this and other comments in terms of how likely these changes are to impact our findings, even though I cannot re-estimate any models without the missing restricted variables.

In our analytic sample, there are only 32 observations (0.5% of the unweighted sample, 0.4% of the weighted sample) who are 18 years old in the first year of data (2013) that would be dropped. It seems reasonable to claim that dropping this small portion of the sample is unlikely to substantively changes in our findings and would hope that the reviewer will allow some leeway given the circumstances. However, we do already have the results of alternate models that exclude 18 to 26 year-olds in the sensitivity analyses presented in the appendix (Tables S4-S6) as that population could have been eligible for dependent coverage through parents if available.

3) The authors made a good choice of excluding those with Medicare from the sample. Additionally, they should also exclude any one with Supplemental Security Income (SSI) as they are eligible for Medicaid as disabled, and the Medicaid program for the disabled have not changed as a result of the ACA.

**AUTHOR RESPONSE**: Similar to our response to the prior comment (Reviewer 1 Comment 2), we unfortunately cannot make this change despite seeing the value in doing so. Approximately 3% of the unweighted analytic sample received any SSI in either year (194 in 2013, 208 in 2014) though this drops to under 2% when weighted (1.7% in 2013 and 1.9% in 2014). Again, it seems reasonable to believe that this small portion of the sample is unlikely to substantively changes our findings and would hope that the reviewer will be understanding given the situation.

**Reviewer 2**

This paper uses linkage between the National Health Interview Survey (NHIS) and the Medical Expenditure Panel Survey (MEPS) to estimate the association between spells of unemployment until 2014 on changes in office-based and ED care in 2014 following insurance expansions from the ACA. The authors do a great job linking these and other datasets to adequately specify their models, and their four category outcome is sufficient to capture changes in treatment setting. Only a few minor points follow.

1) It seems that limited sample size may be the culprit for failing to precisely estimate some effects, particularly the substitution among adults gaining public insurance from long-term uninsurance. The authors state that they use separate regressions for short and long-term uninsured with the same reference group. The authors could consider pooling these treatments and allowing the other controls to be restricted or possibly interact with duration of uninsurance with certain covariates. This may lend some power, although it may not change the findings appreciably while adding some unneeded complexity.

**AUTHOR RESPONSE**: We believe this may have just been a misunderstanding, all of our models include both short- and long-term uninsured along with the continuously enrolled comparison group (see Tables S1, S2, and S3 for the main regression results presented in the manuscript, coefficients for both the short- and long-term uninsured groups are relative to the continuously insured group) to maximize power and enable direct comparability between the groups. We did originally explore breaking duration of uninsurance down into more granular groupings (<6 months, 6-12, 12-23, >=24) but settled upon these because the sample sizes were too small.

2) The public vs. private analyses suffer from having what you can think of as non-compliers to treatment in each sample. Adults with income above 138% FPL cannot take-up public coverage (virtually entirely Medicaid in this case). The authors omit > 400% FPL, but should try also omitting > 138% FPL, sample size permitting. The authors could go further by subsetting to states that expanded Medicaid in 2014 if they are subsetting down to adults who had an opportunity to enroll in Medicaid. Likewise, the private could omit lower income more likely to take-up Medicaid.

**AUTHOR RESPONSE**: We appreciate the suggestion for additional sensitivity analyses and would generally be happy to oblige. As noted above in response to Reviewer 1 Comment 2, all Federal Statistical Research Data Centers are closed indefinitely due to COVID-19 and there is unfortunately no provision for conducting any work virtually given the restricted nature of the data accessible in the RDC. Since these recommendations are for additional sensitivity analyses and not changes to the main results presented in the manuscript, we hope that the reviewer will be understanding about our inability to add these despite otherwise agreeing with the comment.

3) As a minor point, the authors could include as a supplemental table the descriptives for all the variables used in the model, or at least those from the AHRF described in the paper. Also, table notes should include regression sample sizes where applicable.

**AUTHOR RESPONSE**: We have incorporated the county-level variables into Table 1 and added appropriate sample sizes in the footnotes below Tables 2 and 3.
